# Quantum-Dot-Bead-Based Fluorescence-Linked Immunosorbent Assay for Sensitive Detection of Cry2A Toxin in Cereals Using Nanobodies

**DOI:** 10.3390/foods11182780

**Published:** 2022-09-09

**Authors:** Yulou Qiu, Ajuan You, Xianshu Fu, Mingzhou Zhang, Haifeng Cui, Biao Zhang, Weiwei Qin, Zihong Ye, Xiaoping Yu

**Affiliations:** Zhejiang Provincial Key Laboratory of Biometrology and Inspection & Quarantine, College of Life Science, China Jiliang University, Hangzhou 310018, China

**Keywords:** Cry2A, nanobodies, QBs, FLISA

## Abstract

In this study, a quantum-dot-bead (QB)-based fluorescence-linked immunosorbent assay (FLISA) using nanobodies was established for sensitive determination of the Cry2A toxin in cereal. QBs were used as the fluorescent probe and conjugated with a Cry2A polyclonal antibody. An anti-Cry2A nanobody P2 was expressed and used as the capture antibody. The results revealed that the low detection limit of the developed QB-FLISA was 0.41 ng/mL, which had a 19-times higher sensitivity than the traditional colorimetric ELISA. The proposed assay exhibited a high specificity for the Cry2A toxin, and it had no evident cross-reactions with other Cry toxins. The recoveries of Cry2A from the spiked cereal sample ranged from 86.6–117.3%, with a coefficient of variation lower than 9%. Moreover, sample analysis results of the QB-FLISA and commercial ELISA kit correlated well with each other. These results indicated that the developed QB-FLISA provides a potential approach for the sensitive determination of the Cry2A toxin in cereals.

## 1. Introduction

Cry toxins are a group of parasporal crystal proteins produced by *Bacillus thuringiensis* during its sporulation phase, exhibiting excellent insecticidal activity [1,2,3]. At present, its mechanism of action is still unclear. The main proposed mechanism is that Cry toxins combine with the receptors on the midgut cell membrane of insects, forming dissolved pores and resulting in cell lysis, eventually leading to insect death [4,5]. Due to their high toxicity and specificity to insects, Cry-toxin-based insecticides are still the most commonly used biological insecticides since the commercial Cry-toxin-based insecticides were firstly produced in 1938 [6,7]. In addition, Cry toxins are widely introduced into genetically modified (GM) crops for protection against insect pests [8,9]. However, the safety of Cry toxins has not been universally recognized and accepted, and the widespread use of Cry toxins may pose potential threats to the eco-environment and public health [10,11]. Many countries have established mandatory labeling policies for GM products with a threshold level of 0–5% [12]. Therefore, detecting and quantifying Cry toxins in agricultural products and the environment are necessary and important.

Various methodologies, including bioassays, mass spectrometry, surface plasmon resonance (SPR) biosensors, electrochemical immunosensors, real-time PCR, and enzyme-linked immunosorbent assays (ELISAs), have been established for Cry toxins’ analysis [12,13,14,15]. The ELISA methods are appropriate for the on-site determination of Cry toxins with the advantages of being convenient, rapid, and cost efficient [16]. Among them, the most frequently used format is double-antibody sandwich ELISAs (DAS-ELISAs), which generally depend on the traditional polyclonal antibodies (PAbs) and monoclonal antibodies (MAbs) [17,18]. Besides, genetically engineered antibodies (e.g., ScFvs) and phage-displayed peptides have been applied in DAS-ELISA for Cry toxin analysis [19,20]. However, ScFvs and peptides usually exhibited relatively low affinity and poor stability [21,22].

Nanobodies, which were first discovered by Hamers-Casterman in 1993, have attracted much attention in recent years [23,24,25,26]. They are a class of single-domain antibodies derived from heavy-chain antibodies of camelid or related species. Nanobodies are the smallest known functional antibodies, having a low molecular weight of approximately 15 kDa and a small size of 2.5 × 4 nm [27]. Due to their small size and unique structure, nanobodies exhibit many advantageous characteristics, including the ease of manipulation, the ability to recognize inaccessible epitopes, high affinity, and high stability.

Besides, the sensitivity of immunoassays is greatly affected by the signal probes. To date, many fluorescent probes (e.g., quantum dots (QDs), up-conversion nanoparticles, and lanthanide ions) instead of traditional enzymes have been applied to enhance the sensitivity of immunoassays [28,29,30,31,32,33,34,35]. Among them, QDs are ideal fluorescent materials because of their narrow emission spectra, broad excitation, high fluorescent intensity, and high stability [36,37]. In addition, QBs are prepared by embedding plenty of QDs in a polymer matrix, and they show stronger fluorescent intensity than QDs. Li et al. [38] established an immunochromatographic assay using QBs (QBs-ICA) for the detection of cyproheptadine hydrochloride with an IC_50_ of 1.38 ng/mL. The sensitivity of QBs-ICA was 10-times higher than previously report. Qie et al. [39] developed a CdSe/ZnS-QB-based lateral flow immunoassay (LFI) for the T-2 toxin with an LOD of 10 fg/mL, showing an eight-fold higher sensitivity than traditional LFI system.

**Figure 1:**
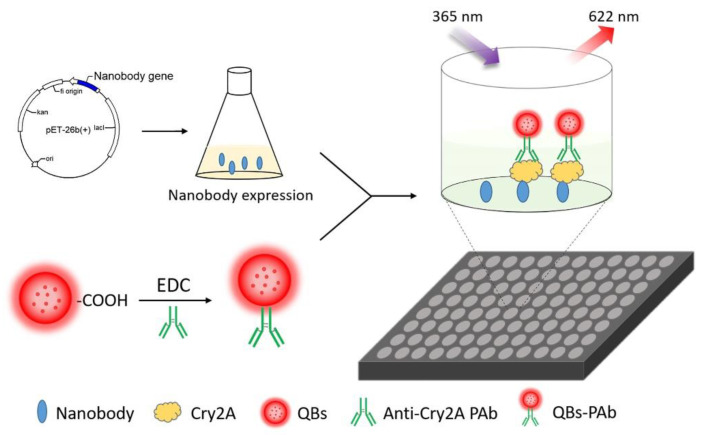
Schematic diagram of the nanobody-based QB-FLISA.

In our previous work, several phage-displayed nanobodies against the Cry2A toxin were isolated from a naive nanobody library [40]. In this study, the anti-Cry2A nanobody P2 was expressed and purified as the capture antibody. QBs were used to couple with the anti-Cry2A PAb to serve as the fluorescent probe. Then, a sensitive QB-based sandwich fluorescence-linked immunosorbent assay (FLISA) using nanobodies was established for the analysis of the Cry2A toxin (Figure 1). The sensitivity and selectivity of the established assay were investigated. In addition, the QB-FLISA and commercial ELISA kit were used to detect the Cry2A toxin in cereal samples. The established QB-FLISA has the potential to be a reliable method for detecting the Cry2A toxin in cereals.

## 2. Materials and Methods

### 2.1. Chemicals and Reagents

Cry toxins (Cry2A, Cry1Ab, Cry1B, and Cry3Bb) and the Cry2A commercial ELISA kit were purchased from You Long Bio. Co., Ltd. (Shanghai, China). QBs were purchased from Kundao (Shanghai, China). Phage-displayed nanobody P2 and anti-Cry2A PAb were prepared previously [40]. HRP-labeled goat anti-rabbit antibody was purchased from Solarbio (Beijing, China). Restriction enzymes *Not* I, *Nco* I, T4 DNA ligase, and TaqDNA polymerase were purchased from Takara Co. (Dalian, China). Isopropylthio-β-D-galactoside (IPTG), 96-well microplates, and nickel-nitrilotriacetic acid (Ni-NTA) resin were purchased from Sangon Biotech (Shanghai, China). Bovine serum albumin (BSA) was purchased from Sigma-Aldrich (St. Louis, MO, USA). The TMB substrate was purchased from Beyotime (Shanghai, China).

### 2.2. Expression and Purification of Anti-Cry2A Nanobodies

A gene fragment of the anti-Cry2A nanobody P2 was amplified by PCR (94 °C for 3 min, followed by 30 cycles of 94 °C for 30 s, 55 °C for 30 s, and 72 °C for 30 s) and digested by restriction enzymes *Not* I and *Nco* I. Then, the gene fragment of the nanobody P2 was gel purified and inserted into the pET-26b(+) vector. The recombinant vectors were transferred into *E. coli* DH5α-competent cells. After confirmation was achieved by PCR and sequencing, the target recombinant vector was transferred into *E. coli* Rosetta (DE3). The recombinant *E. coli* strains were spread on the LB plate supplemented with 40 μg/mL kanamycin, and individual colonies were incubated in LB liquid medium by shaking overnight. Then, the cultured bacteria were inoculated into 100 mL of fresh LB-kanamycin liquid culture and incubated to an OD600 of 0.5. The recombinant cells were induced with IPTG at 0.2 mM by shaking (200 rpm) for 12 h and harvested by centrifugation (8000× *g*, 15 min). Subsequently, the cell suspensions were broken by sonication and separated by centrifugation (10,000× *g*, 15 min). Then, supernatants were collected and filtered through a 0.22 μm membrane. The soluble nanobody with a 6 × His-tag was purified by the Ni-NTA affinity column. The purified nanobodies were confirmed by SDS-PAGE in accordance with the standard protocol. The concentration of purified nanobodies was measured using a Nanodrop 1000 (Thermo, Waltham, MA, USA).

### 2.3. Preparation of QBs-PAb

QBs-PAb conjugates were synthesized according to the published procedure with some modification [41]. In brief, 10 μL of QBs (10 mg/mL) and 5 μL of EDC (10 mg/mL) were added to 500 μL of PB buffer (10 mM, pH 6.0) and mixed at RT for 30 min. After these reaction mixtures were separated by centrifugation (10,000× *g*, 10 min), the precipitate was collected and resuspended in 400 μL PB buffer. Subsequently, 50 μL of anti-Cry2A PAbs (2.0 mg/mL) was added into the buffer and mixed at RT for another 30 min. Afterwards, the mixture was blocked by 1% BSA solution for 1 h incubation. After centrifugation (10,000× *g*, 10 min), the precipitate was resuspended in 10 mM PBS buffer with 1% BSA and 0.5% Tween-20. Finally, the QBs-PAb conjugates were stored at 4 °C for further use. Transmission electron microscopy (TEM) images were recorded by an electron microscope (JEOL, Tokyo, Japan) operated at 200 kV. Fluorescence spectra (excitation at 365 nm) were recorded with a fluorescence microplate reader (Tecan, Männedorf, Switzerland). Dynamic light scattering (DLS) analysis was carried out on a particle size analyzer (Malvern Instruments, Malvern, UK).

### 2.4. QBs-Based FLISA

Using nanobodies as the capture antibody, a double-antibody sandwich FLISA (DAS-FLISA) based on QBs for detecting the Cry2A toxin was developed. First, the nanobody P2 (5.0 μg/mL, 100 μL/well) was added to the black microplate. After incubation overnight at 4 °C, the plates were washed three times in 0.05% PBST, and the excess binding sites were blocked for 2 h in blocking buffer (3% BSA in 0.1% PBST). Subsequently, the microplate was washed three times with 0.05% PBST, and different concentrations of Cry2A toxins (0–1000 ng/mL, 100 μL/well) were added to the washed wells for incubation at 37 °C for 1 h. Next, QBs-PAb probes were added to the microplate and incubated for 1 h. Finally, unbound QBs-PAb probes were discarded by washing six times, and the fluorescence intensity of each well was measured by a multimode microplate reader (Tecan, Männedorf, Switzerland). The working concentrations of the nanobody P2 and QBs-PAb were optimized in advance to obtain the best performance of the QB-FLISA.

### 2.5. Cross-Reactivity

The specificity of the QB-FLISA was evaluated against the Cry1Ab, Cry1B, and Cry3Bb toxins. Briefly, 100 μL of the nanobody P2 per well was added to black microtiter plates and incubated overnight at 4 °C. After the plates were blocked with blocking buffer and washed thrice with 0.05% PBST, 100 μL of different Cry toxins (1000 ng/mL) was added into the microtiter plates for 1 h incubation. The following steps were the same as those described above. Each assay was performed in three replicates for each toxin.

### 2.6. Analysis of Spiked Cereal Samples

The accuracy and precision of the established QB-FLISA were estimated by calculating the recoveries and coefficients of variation of the intra- and inter-assays. Intra-assays were conducted within 1 day in three replicates on each spiked sample. Inter-assays were performed in three replicates on each sample once per day for 3 consecutive days. Cry2A-free cereal samples (corn and rice) were collected from a local farm (Hangzhou, China). The blank cereal sample was spiked with the Cry2A toxin at different concentrations of 200, 1000, and 5000 ng/g. The cereal sample extraction procedure was operated according to the reported method with slight modifications [42]. In brief, 5.0 g of the cereal sample was crushed by a blender. Then, 1.0 g of the cereal sample was weighed and added into 2 mL of extraction buffer (0.2% BSA in 0.1% PBST). After the mixture was gently shaken for 30 min at RT, it was separated by centrifugation (10,000× *g*, 15 min). The supernatant was collected and further diluted 10 times with extraction buffer for QB-FLISA analysis.

## 3. Results and Discussion

### 3.1. Preparation and Characterization of the Anti-Cry2A Nanobody

**Figure 2:**
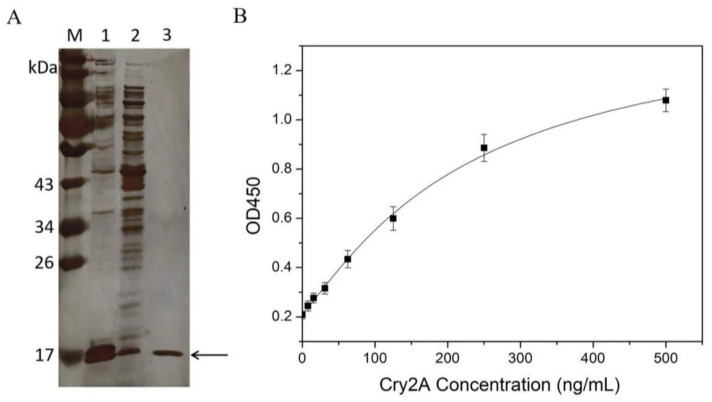
(**A**) SDS-PAGE analysis of the nanobody P2. Lane M: protein marker; Lane 1: the supernatant proteins of the induced *E. coli* cells after sonication; Lane 2: the precipitated protein of the induced *E. coli* cells after sonication; Lane 3: the purified nanobody P2. (**B**) Standard curve (logistic fit) for the Cry2A toxin in the nanobody-based DAS-ELISA. The error bars represent the standard deviation (*n* = 3).

Recombinant expression vector pET-26b(+)-P2 was constructed and confirmed by colony PCR and sequencing. Then, the positive recombinant vector was transformed into *E. coli* Rosetta (DE3). The protein of the nanobody P2 was expressed by IPTG induction and purified on a Ni-NTA affinity chromatography. Subsequently, the expressed and purified nanobody was characterized by SDS-PAGE. Figure 2A shows a target band around 17 kDa observed in the induced *E. coli* cells. The amount of nanobody expressed in the supernatant was obviously higher than that expressed in the inclusion body. The purity of the purified nanobody P2 was over 90%, and the expression yield of the nanobody in the LB culture medium was approximately 30 mg/L.

### 3.2. Nanobody-Based DAS-ELISA

The purified nanobody P2 was applied to establish a DAS-ELISA for Cry2A toxin analysis. Briefly, 100 μL of the purified nanobody P2 (3.0 μg/mL in PBS) was added into microtiter plates and coated at 4 °C overnight. After blocking was conducted with blocking buffer (3% BSA in 0.1% PBST), serial concentrations of the Cry2A toxins (0–500 ng/mL, 100 μL/well) were added to the microplate and incubated at 37 °C for 1 h. Next, the anti-Cry2A PAb was transferred into the microplate for 1 h of incubation. Then, the plate was washed five times with 0.05% PBST, and 100 μL per well of HRP-labeled goat anti-rabbit antibody was transferred into the plate for another 1 h incubation. Finally, TMB substrates (100 μL/well) were added and incubated for 15 min at 37 °C. The OD450 values were detected by a microtiter plate reader. The results showed that the OD450 value increased with the increase in the concentration of the Cry2A toxin, indicating the good binding activity of the nanobody P2. Meanwhile, a standard curve of the DAS-ELISA was constructed, and the low detection limit (LOD) of the method, calculated as three-times the standard deviation of the blank signal, was 7.83 ng/mL (Figure 2B).

In general, the establishment of the DAS-ELISA depends on conventional MAbs and PAbs. However, the MAb preparation process is complicated and needs a long production period (5–6 months), whereas PAbs cannot achieve the demand of large batches and continual production [43]. Several recognition elements (e.g., nanobodies, peptides, and aptamers), which have emerged in recent years, have been used as substitute for conventional antibodies for the development of immunoassays. Nanobodies can be isolated from a naive phage-displayed nanobody library and further expressed and purified within two weeks, greatly improving the preparation efficiency of antibodies. In addition, nanobodies have excellent characteristics, including high affinity, stability, solubility, and reproducibility, which make them a promising reagent in immunoassays.

### 3.3. Characterization of QBs-PAb Conjugates

**Figure 3:**
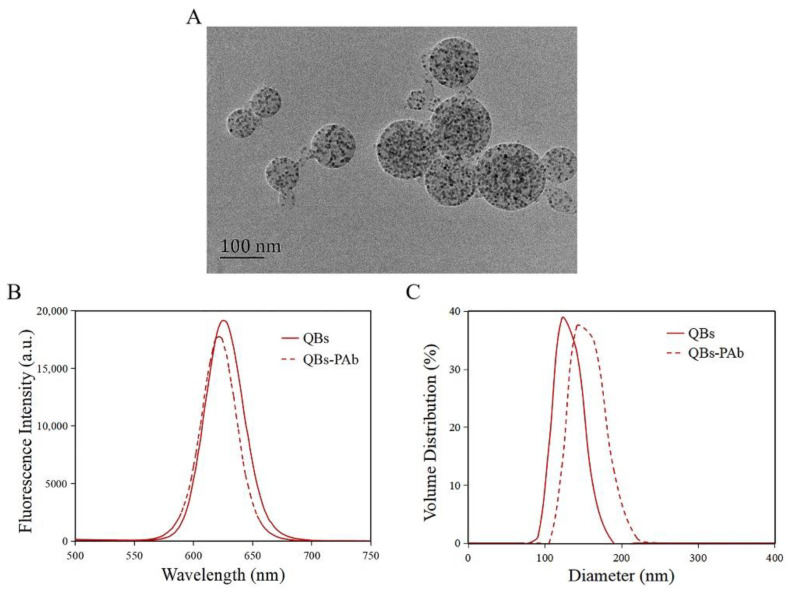
Characterization of QBs and QBs-PAb. (**A**) TEM image of the QBs. (**B**) Fluorescence spectroscopy of QBs and QBs-PAb. (**C**) Hydrodynamic diameter of QBs and QBs-PAb.

The morphology of the QBs was characterized by TEM. As shown in Figure 3A, the QBs were round and distributed uniformly, with numerous QDs encapsulated in the polymer matrix. The QBs-PAb probe was characterized by fluorescence spectra and DLS analysis. The results showed that the maximum emission wavelength of QBs was 626 nm, while that of the QBs-PAb exhibited a slight blue shift to 622 nm (Figure 3B). In addition, the fluorescence intensity of the QBs-PAb probe slightly decreased compared with that of the QBs, indicating that the PAb modification did not significantly affect the fluorescence emission properties of the QBs. The particle size analysis of the QBs and QBs-PAb is presented in Figure 3C. The average hydrodynamic diameter of the QBs-PAb (154 nm) was larger than the diameter of the QBs (129 nm), indicating that the PAb was coupled on the surface of the QBs. Therefore, these results demonstrated that the QBs-PAb fluorescent probe was successfully synthesized.

### 3.4. Development of QB-FLISA

**Figure 4:**
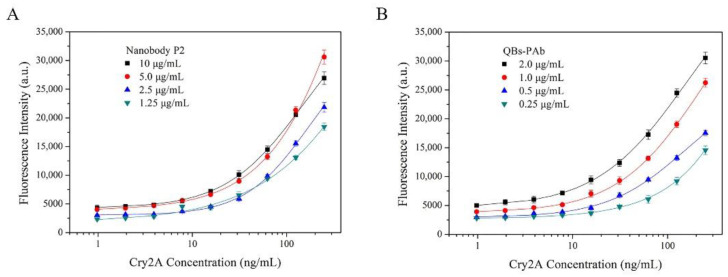
Optimization of nanobody-based QB-FLISA. (**A**) Optimization of the concentrations of the nanobody P2 (10, 5.0, 2.5, and 1.25 μg/mL). (**B**) Optimization of the concentrations of the QBs-PAb (2.0, 1.0, 0.5 and 0.25 μg/mL).

**Figure 5:**
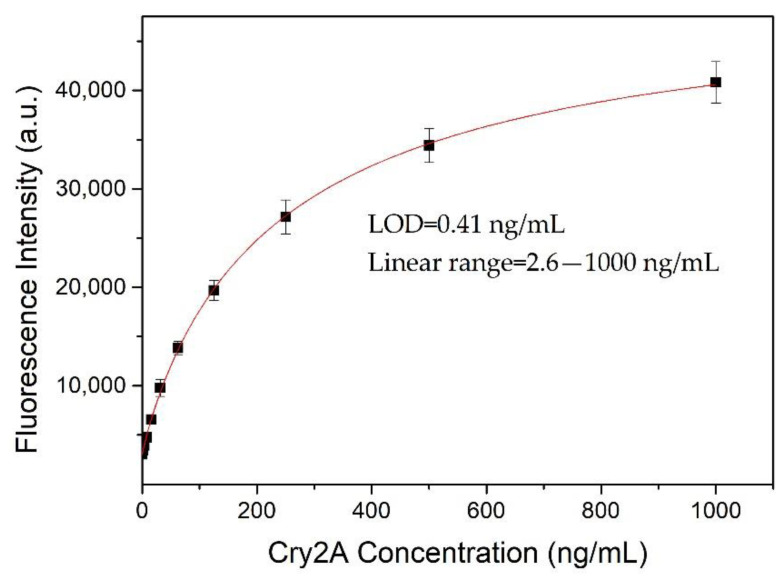
Standard curve (logistic fit) of nanobody-based QB-FLISA for Cry2A toxin analysis under optimized conditions. The error bars represent the standard deviation (*n* = 3).

A nanobody-based QB-FLISA was established for the determination of Cry2A. The nanobody P2 was used as the capture antibody, and the QB-labeled anti-Cry2A PAb served as the detection probe. Different concentrations of the nanobody P2 (10, 5.0, 2.5, and 1.25 μg/mL) and QBs-PAb (2.0, 1.0, 0.5 and 0.25 μg/mL) were optimized to improve the performance of the proposed QB-FLISA. The LOD value and signal-to-noise ratio were used to evaluate the assay’s performance. As shown in Figure 4A, the assay’s performance was similar in the P2 concentrations of 10 and 5.0 μg/mL, so the optimal concentration of the nanobody P2 was determined to be 5.0 μg/mL. The fluorescent intensity reduced as the concentration of the QBs-PAb decreased from 2.0 μg/mL to 0.25 μg/mL (Figure 4B). Thus, the concentration of 2.0 μg/mL was selected as the optimal concentration of the QBs-PAb. Subsequently, a nanobody-based QB-FLISA for Cry2A was developed under the optimum experimental conditions. As shown in Figure 5, the developed nanobody-based QB-FLISA exhibited good linearity in the range of 2.6–1000 ng/mL. The LOD value calculated as three-times the standard deviation of the blank signal was 0.41 ng/mL, which was 19-times lower than conventional colorimetric ELISA. This finding indicated that QBs are a favorable fluorescent marker for improving sensitivity in immunoassays for the Cry2A toxin. Comparisons of the proposed QB-FLISA with previously reported methods revealed that the QB-FLISA exhibited a good sensitivity and linearity for Cry2A analysis (Table 1).

### 3.5. Cross-Reactivity

**Figure 6:**
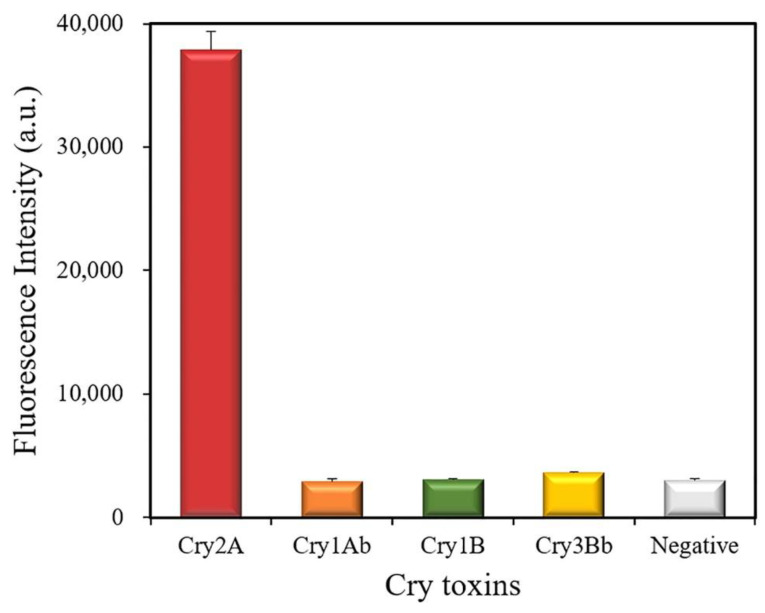
Cross-reactivity of the QB-FLISA with the other Cry toxins (1000 ng/mL).

The cross-reactivity of the developed QB-FLISA was estimated against other Cry toxins (1000 ng/mL), including Cry1Ab, Cry1B, and Cry3Bb (Figure 6). The results showed a strong fluorescent intensity at 622 nm measured for the Cry2A toxin, but no positive signal was found for the other Cry toxins (Cry1Ab, Cry1B, and Cry3Bb), indicating that the proposed method has no cross-reactivity with the other Cry toxins (1000 ng/mL). These results indicated the good specificity of the developed nanobody-based QB-FLISA for the Cry2A toxin.

### 3.6. Sample Analysis and Validation

**Figure 7:**
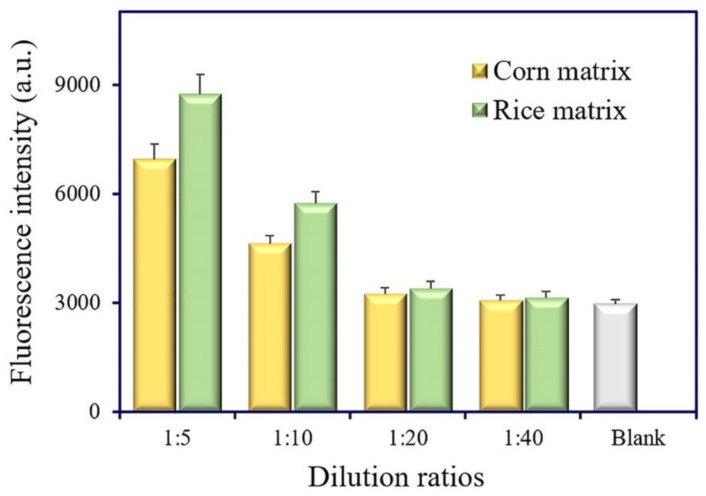
Estimation of the matrix effect in corn and rice samples.

Matrix interference is a universal phenomenon in real sample analysis. The dilution of sample extracts is a simple and practical method to eliminate matrix interference. The matrix effect of cereal samples (corn and rice) was estimated at different dilution ratios (1:5, 1:10, 1:20, and 1:40). No evident difference was observed between the 20-fold dilution of extracts and the extraction buffer (Figure 7). Thus, the 20-fold dilution of extracts was selected and used for cereal samples’ (corn and rice) analysis.

A spike-and-recovery experiment was conducted to estimate the accuracy and precision of the developed nanobody-based sandwich QB-FLISA. The Cry2A-free cereal samples (corn and rice) spiked with different concentrations of Cry2A toxin (200, 1000, and 5000 ng/g) were tested. As shown in Table 2, the average recoveries of Cry2A in the corn sample ranged from 89.6% to 117.3%, with a coefficient of variation (CV) in the range of 5.5–7.7%. Meanwhile, the recovery in the rice sample ranged from 86.6–109.5%, with a CV in the range of 6.2–8.2%. The Cry2A commercial ELISA kit was used to validate the results of the QB-FLISA. Overall, the results from the two methods correlated well with each other. These results demonstrated that the proposed QB-FLISA is applicable for the detection of Cry2A in cereal samples.

## 4. Conclusions

In this work, an anti-Cry2A nanobody P2 was prepared and applied to develop a DAS-FLISA that was based on QBs for sensitive detection of the Cry2A toxin. The linearity range of the developed nanobody-based QB-FLISA was determined to be 2.6–1000 ng/mL. The LOD of the method was 0.41 ng/mL, which exhibited a 19-fold higher sensitivity compared to the traditional colorimetric ELISA. The spike-and-recovery experiment results showed that the developed method achieved acceptable recoveries (86.6–117.3%) and CVs (5.5–8.2%) for Cry2A detection in cereal samples. These results demonstrated that QBs could be a favorable probe for improving sensitivity in immunoassays. The proposed nanobody-based QB-FLISA may have potential for the sensitive detection of the Cry2A toxin in cereals.

## Figures and Tables

**Table 1:** Comparison of some published methods for Cry toxins’ detection.

Detection Method	Antibody	LOD	Working Range	Cry Toxin	Matrix	Reference
Colorimetric ELISA	Traditional antibody	0.27–0.51 ng/mL	0.45–15.71 ng/mL	Cry 1Ie	maize	[2]
Colorimetric IC-ELISA	Human domain antibody	0.029–0.074 μg/mL	0.258–1.407 μg/mL	Cry1Ab, Cry1Ac, Cry1B, Cry1C, and Cry1F	wheat	[8]
Electrochemiluminescent immunosensor	Traditional antibody	3.0 pg/mL	0.010–1.0 ng/mL	Cry1Ab	rice and maize	[10]
Colorimetric ELISA	Traditional antibody	0.47 ng/mL	2.5–100 ng/mL	Cry1Ab	rice	[16]
Colorimetric ELISA	Traditional antibody	15 ng/mL	0.015–32 μg/mL	Cry1	–	[18]
Colorimetric ELISA	ScFv	4.6–9.2 ng/mL	12–250 ng/mL	Cry1Aa, Cry1Ab, and Cry1Ac	–	[19]
Colorimetric ELISA	Phage-displayed peptide	8 ng/mL	10–50.625 ng/mL	Cry2Ad2-3	corn	[20]
QB-FLISA	Nanobody	0.41 ng/mL	2.6–1000 ng/mL	Cry2A	corn and rice	This work

**Table 2:** Recoveries of Cry2A toxin spiked in cereal samples by nanobody-based QB-FLISA.

Matrix	Spike Level (ng/g)	Intra-Assay (*n* = 3)			Inter-Assay (*n* = 3)			Commercial ELISA Kit (*n* = 3)		
		Mean ± SD (ng/g)	Recovery (%)	CV (%)	Mean ± SD (ng/g)	Recovery (%)	CV (%)	Mean ± SD (ng/g)	Recovery (%)	CV (%)
Corn	200	231.2 ± 16.6	115.6	7.2	223.5 ± 15.7	117.3	7.0	222.8 ± 15.2	111.4	6.8
	1000	962.4 ± 52.8	96.2	5.5	941.1 ± 64.5	94.1	6.9	976.2 ± 57.5	97.6	5.9
	5000	4545 ± 327	90.9	7.2	4480 ± 346	89.6	7.7	4525 ± 371	90.5	8.2
Rice	200	210.8 ± 16.5	105.4	7.8	221.9 ± 18.3	109.5	8.2	233.8 ± 20.4	116.9	8.7
	1000	1027 ± 76.2	102.7	7.4	1018 ± 76.1	101.8	7.5	1082 ± 72.6	108.2	6.7
	5000	4330 ± 269	86.6	6.2	4412 ± 301	88.2	6.8	4635 ± 329	92.7	7.1

## Data Availability

The data presented in this study are available on request from the corresponding author.
